# Poor knowledge of methotrexate associated with older age and limited English-language proficiency in a diverse rheumatoid arthritis cohort

**DOI:** 10.1186/ar4340

**Published:** 2013-10-22

**Authors:** Jennifer L Barton, Gabriela Schmajuk, Laura Trupin, Jonathan Graf, John Imboden, Edward H Yelin, Dean Schillinger

**Affiliations:** 1 Department of Medicine, Division of Rheumatology, University of California, San Francisco, 3333 California Street, Box 0920, San Francisco, CA 94143, USA; 2 UCSF Center for Vulnerable Populations, San Francisco General Hospital, San Francisco, CA, USA

## Abstract

**Introduction:**

Our objective was to determine rheumatoid arthritis (RA) patients’ understanding of methotrexate and assess whether knowledge varies by age, education, English language proficiency, or other disease-related factors.

**Methods:**

Adults with RA (n = 135) who were enrollees of an observational cohort completed a structured telephone interview in their preferred language between August 2007 and July 2009. All subjects who reported taking methotrexate were asked 11 questions about the medication in addition to demographics, education level, and language proficiency. Primary outcome was a total score below the 50^th^ percentile (considered inadequate methotrexate knowledge). Bivariable and multivariable logistic regressions were performed. Covariates included demographics, language proficiency, education, and disease characteristics.

**Results:**

Of 135 subjects, 83% were female, with a mean age of 55 ± 14 years. The majority spoke English (64%), followed by 22% Spanish and 14% Cantonese or Mandarin. Limited English language proficiency (LEP) was reported in 42%. Mean methotrexate knowledge score was 5.4 ± 2.6 (range, 0 to 10); 73 (54%) had a score lower than 5 (of 10). Age older than 55, less than high school education, LEP, better function, and biologic use were independently associated with poor knowledge.

**Conclusions:**

In a diverse RA cohort, overall methotrexate knowledge was poor. Older age and limited proficiency in English were significant correlates of poor knowledge. Identification of language barriers and improved clinician-patient communication around methotrexate dosing and side effects may lead to improved safety and enhanced benefits of this commonly used RA medication.

## Introduction

Methotrexate (MTX) is a recommended first-line treatment for rheumatoid arthritis (RA) [1,2], with up to 30% of patients achieving an American College of Rheumatology 70% response (ACR70) or better when taking MTX alone. Despite its efficacy, MTX is associated with adverse events in >70% of patients [3], even though the majority continue to take it. In a systematic review of RA patients who had taken MTX for 2 years or more, adverse events led to discontinuation of MTX in one-third of patients [3]. Poor understanding by patients of the side effects and potential toxicities of methotrexate may increase the incidence of MTX-associated adverse effects and thus limit use of MTX or cause unnecessary harm.

In the United Kingdom and Europe, recommendations for improving the safety of MTX use include patient education. However, no similar guideline exists in the United States, where it has been shown that 90 million adults have limited health literacy [4], and an ever-growing population lacks English language proficiency (8.1% of the U.S. population age 5 years and older, 2000 US Census data). To date, no U.S. studies of methotrexate knowledge among vulnerable populations with RA and limited English language proficiency have been conducted.

Therefore, we sought to determine RA patients’ basic understanding of MTX among a diverse cohort of adults and to assess whether knowledge varied by age, education level, English language proficiency, or other disease-related factors.

## Methods

### Data source

The data source was the University of California, San Francisco (UCSF) RA cohort, a dual-site observational cohort. Beginning in October 2006 [5,6], established patients were consecutively enrolled from two clinics staffed by UCSF faculty and fellows: the RA clinic at a county hospital and a university-based arthritis clinic. At time of enrollment, patients had to be age ≥18 years and meet the 1987 American College of Rheumatology criteria for RA [7]. The research protocol was approved by the UCSF Committee on Human Research, and all participants provided informed consent to participate in the study as well as consent to publish results.

Subjects included in the present study completed a structured telephone survey in their preferred language of English, Spanish, Cantonese, or Mandarin, and reported the use of methotrexate at the time of the telephone interview. Enrollment in the telephone survey was conducted by recruiting RA cohort members as they appeared for regularly scheduled follow-up visits at the two clinics, between August 2007 and July 2009. For the Chinese and Spanish survey instruments, we used a translation service accredited by the American Translation Association that works exclusively with native speakers and uses an iterative translation-back translation process. Of the 244 subjects interviewed by telephone, 135 reported taking methotrexate and thus were eligible to be included in this study. Subject characteristics of those not taking methotrexate did not differ from those who reported taking methotrexate by age, gender, race, language, education, immigrant status, biologic use, or clinic site. Subjects who did not report MTX use did have longer disease duration (14 ± 13 years versus 9 ± 10 years; *P* = 0.001) and worse disability (HAQ, 1.35 ± 0.81 versus 1.06 ±0.85; *P* = 0.008).

### Primary outcome: inadequate methotrexate knowledge

Subjects who reported taking methotrexate at the time of the telephone survey were asked 11 questions about the medication. The methotrexate questions were adapted from a previously validated Methotrexate Knowledge Questionnaire published in 1996 [7], with two new questions added regarding the role of folic acid and the mechanism of action (see Additional file 1 for full questionnaire). We excluded the question about birth defects from the full score, as it was not relevant for women past childbearing age (50 years or older). Thus, each individual had a total score on the questionnaire equal to the number of correct answers (of 10). The primary outcome was a total knowledge score below the 50^th^ percentile (5 points), which we considered inadequate methotrexate knowledge.

### Secondary outcomes

The secondary outcomes were a correct response to individual questions about the following: birth defects among women younger than 50 years and men, methotrexate dosing, and alcohol use. Two additional questions that assessed numeracy related to methotrexate were also included in the questionnaire (Additional file 1). These two questions were analyzed separately, as they captured the domain of numeracy more than actual knowledge about the medication.

### Primary predictors

We anticipated that younger age, English language proficiency, and higher education would be associated with methotrexate knowledge scores. Age was dichotomized at the median, 55 years. English language proficiency was assessed by using the U.S. Census question “How well do you speak English?” Those who reported “very well” were considered English proficient (EP), and those who reported “well,” “not well,” or “not at all” were considered to have limited English proficiency (LEP) [8]. Education level was ascertained during the telephone interview and dichotomized as less than high school (<HS) or high-school graduate and beyond (HS/BA).

### Other covariates

The subjects’ race/ethnicity, sex, disease duration, and clinic site were recorded at enrollment. Measurement of RA disease characteristics included disease duration, biologic use, and function, as captured by the 20-item Health Assessment Questionnaire (HAQ) [9]. Biologic use, as reported by the patient, was recorded by the physician in the chart at each visit. Biologic disease-modifying antirheumatic drugs (DMARDs) included etanercept, infliximab, adalimumab, rituximab, and abatacept.

### Statistical analysis

We compared methotrexate knowledge scores between patients with LEP and EP, <HS and HS/BA education, age younger than 55 years and 55 years or older by using *t* tests. We then conducted bivariable and multivariable logistic regressions of poor methotrexate knowledge (score below the 50^th^ percentile). Predictors included demographics (age, gender, race/ethnicity, language, education), English-language proficiency, and disease characteristics (disease duration, HAQ, biologic use). Given the significant collinearity between education and English-language proficiency, we developed two multivariable models, one including education and one with LEP in place of education. All other demographic and disease characteristics were included in both models, with the exception of race and language. Race was not significantly associated with poor MTX knowledge in bivariate analyses and, given significant the collinearity of language with LEP, these were not included in the final models. As a sensitivity analysis, we added the numeracy questions to the 10-item questionnaire and ran both models 1 and 2. Individual questions on birth defects, frequency of dosing and alcohol (ETOH) were also analyzed separately in both bivariable and multivariable analyses. Internal validity of the questionnaire was assessed by using Cronbach’s alpha. No systematic, formal methotrexate teaching is done by rheumatology faculty and fellows, nurses, or pharmacists across the two clinics. STATA SE 9.2 was used for all analyses.

## Results

Of the 135 subjects who reported taking methotrexate and completed the knowledge questionnaire, 83% were female, with a mean (±SD) age of 55 ± 14 years (Table 1). The diversity of the UCSF RA Cohort was reflected in this sample with 32% white, 33% Latino, 23% Asian/Pacific Islander, 9% African American, and 4% other. The majority of subjects spoke English (64%), followed by 22% Spanish and 14% Cantonese or Mandarin. Limited English-language proficiency was reported in 42%, and less than high school education was reported by 28%. Mean (± SD) disease duration was 9 ± 10 years, and mean (± SD) HAQ score was 1.1 ± 0.8 (range, 0 to 3). Forty-four percent of subjects reported taking a biologic DMARD in addition to methotrexate.

**Table 1:** Demographics of 135 methotrexate users

**Characteristic**	**Total *N*** **= 135**
	Mean ± SD
Age, years	55 ± 14
Disease duration, years	9 ± 10
HAQ score	1.06 ± 0.85
	N (%)
Female	112 (83)
Race/ethnicity	
Non-Hispanic White	43 (32)
Latino	44 (33)
Asian/Pacific Islander	31 (23)
African American	12 (9)
Other	5 (4)
Language	
English	86 (64)
Spanish	30 (22)
Chinese	19 (14)
Non-U.S. born	71 (53)
University clinic	71 (53)
Public hospital clinic	64 (47)
Less than high school education	38 (29)
Limited English language proficiency	57 (42)
Biologic use	59 (44)

The responses to each individual item on the methotrexate knowledge score are shown in Table 2. At least 80% of subjects correctly answered questions on once-weekly dosing, need for frequent monitoring (84%), and (among women younger than 50 years) risk of birth defects while taking methotrexate (81%). Two thirds of patients were aware of the disease-modifying aspect of methotrexate as well as the purpose of folic acid. Specific questions for which knowledge was inadequate were the risk of birth defects among men (22% correct), alcohol consumption, and other potential side effects. In particular, cytopenias or lung hypersensitivity was identified by only 27% and 23% of participants, respectively. With regard to the two numeracy items that were not included in the total methotrexate knowledge score, 66% and 60% answered correctly.

**Table 2:** Percentage correct on methotrexate knowledge items of 135 RA subjects who reported taking methotrexate

	**Percentage correct**
1. Once-weekly dosing	80
2. Alcohol consumption	53
3. Frequency of blood-test monitoring	84
4a. Teratogenicity (women <50)	81
4b. Teratogenicity (men)	22
Possible side effects	
5. Nausea	42
6. Cytopenias	27
7. Stomatitis	43
8. Hypersensitivity pneumonitis	23
9. Hepatotoxicity	57
10. Role of folic acid	64
11. Methotrexate as disease modifier	64
12. Numeracy: total weekly dose	66
13. Numeracy: split dose on same day	60

**Figure 1:**
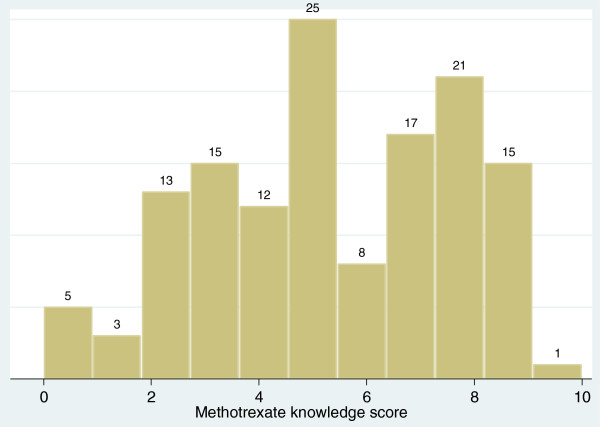
**Distribution of methotrexate knowledge scores among 135 subjects.** Mean knowledge score was 5.4 ± 2.6; median score was 5 (range, 0 to 10). Numbers above each column reflect the number of patients with that score.

The mean (±SD) methotrexate knowledge score was 5.4 ± 2.6 (range, 0 to 10); 73 (54%) participants had a score lower than 5 (of 10). The distribution of scores is shown in Figure 1. Cronbach’s alpha coefficient for the 10-item scale was 0.75 and, for the 12-item (which included the numeracy questions), was 0.76. The most significant difference in scores was seen by age, with a mean score of 5.9 ± 2.7 for those 55 or younger compared with a score of 4.9 ± 2.4 for subjects older than 55 years (*P* = 0.03). No significant differences were found by gender, race/ethnicity, or disease duration (data not shown).

To identify potential correlates of poor methotrexate knowledge, we conducted logistic regression on the likelihood of MTX knowledge scores below the 50^th^ percentile (5 of 10; Table 3). In bivariate analyses, age older than 55 was correlated with poor methotrexate knowledge (*P* = 0.023). The association of older age with poor methotrexate knowledge remained significant when adjusting for gender and education, as well as disease characteristics. Less than high school education was also associated with poor knowledge (adjusted odds ratio, 3.68; 95% CI, 1.42 to 9.54). Disease characteristics associated with poor methotrexate knowledge included biologic use (AOR, 3.28; 95% CI, 1.40 to 7.65) and better function as measured by HAQ (AOR, 0.55; 95% CI, 0.34 to 0.90).

**Table 3:** Predictors of methotrexate knowledge, with and without adjustment for demographic and disease characteristics, limited English language proficiency, and biologic use

	**Unadjusted OR (95% confidence interval)**	**Adjusted OR (95% confidence interval)**	
**Predictor**		**Model 1**	**Model 2**
Age older than 55 years	2.23 (1.11, 4.43)	3.29 (1.48, 7.30)	3.08 (1.40, 6.79)
Female gender	0.89 (0.34, 2.19)	0.69 (0.23, 2.07)	0.87 (0.30, 2.50)
Less than high school education	1.96 (0.90, 4.29)	3.68 (1.42, 9.55)	-
Limited English language Proficiency	1.90 (0.95, 3.82)	-	2.60 (1.14, 5.90)
Disease duration, per year	0.98 (0.93, 1.01)	0.97 (0.93, 1.01)	0.97 (0.93, 1.01)
HAQ	0.74 (0.49, 1.12)	0.55 (0.34, 0.90)	0.61 (0.38, 0.97)
Biologic use, yes	1.87 (0.93, 3.74)	3.28 (1.41, 7.65)	3.02 (1.31, 7.00)

Results in models 1 and 2 are adjusted for all variables shown.

In a second multivariate analysis (model 2) to assess the association of LEP with methotrexate knowledge, we replaced education with LEP (as the two were highly correlated) and noted a significant association of limited English language proficiency with poor knowledge (AOR, 2.13; 95% CI, 1.03 to 4.41). Age older than 55 years, biologic use, and better HAQ all remained significant in this model. In a sensitivity analysis, which included both of the numeracy items in the total score (50^th^ percentile, 7 of 12), all predictors that were significant in the original models 1 and 2 remained significant, with the exception of HAQ, although the direction of the association was the same (*P* = 0.06; 0.15, respectively, for models 1 and 2).

Separate analyses were run for the ETOH item and then for the birth-defect question, including only women of childbearing age and men, as these were deemed critical pieces of knowledge. Older age and taking a biologic were associated with poor knowledge about ETOH consumption. Education was not a significant correlate, whereas limited English proficiency was associated with a greater likelihood of correctly identifying the risk of ETOH consumption (AOR for an incorrect response: AOR, 0.32; 95% CI, 0.15 to 0.70). This association was the opposite of that in the original model. In separate analyses of the birth-defect question, female gender and longer disease duration were associated with better knowledge in both models (results not shown).

## Discussion

Among a diverse cohort of adults with rheumatoid arthritis taking MTX, knowledge of certain aspects of methotrexate was high, such as once-weekly dosing, teratogenicity among women of childbearing age, and toxicity monitoring. Knowledge was poor, however, among men regarding potential for birth defects and for all subjects of potential side effects. Age older than 55, limited English-language proficiency, lack of education, and biologic use were independently associated with poorer methotrexate knowledge.

Methotrexate is the cornerstone of therapy in rheumatoid arthritis, and knowledge of dosing, side effects, teratogenicity, and monitoring are essential to its safe and effective use. In addition to the safety aspects of informing patients, clinicians should be educated about and encouraged to follow national and international guidelines [10] to involve patients in decision making around rheumatoid arthritis medications and fully inform patients and caregivers of the comparative harms and benefits of disease-modifying agents. The majority of RA patients in the United States are prescribed methotrexate by a rheumatologist and continue to be seen by the subspecialist in follow-up. However, in the UK, patients more often receive their initial prescription from the rheumatologist but then go on to have all refills and monitoring by a general practitioner (GP). One study in the UK evaluated GP knowledge of monitoring for patients taking MTX and found that only 58% were aware of local monitoring guidelines, and 48% were aware of national guidelines [11]. A uniform, systematic approach to patient education delivered in plain language, available in multiple languages and formats, with periodic reinforcement, should be standard of care along with a basic minimum standard of MTX knowledge on the part of the provider.

It is critical to identify those patients at higher risk of poor understanding and suboptimal communication with their clinician, such as those with limited English-language proficiency and/or low education. We showed in prior work that patients with RA who are not English speakers and who are immigrants have poorer outcomes of higher disease activity by the Disease Activity Score-28 and poorer function, even when adjusting for DMARD use, age, education, and disease characteristics [12]. These patients are at high risk for poorer outcomes and herein were shown to have poorer knowledge of one of the most commonly used DMARDs for RA. Although it is known that language barriers have adverse effects on access to care, quality of care, patient satisfaction, and outcomes in other chronic diseases [13], little work has been done to assess variation in knowledge of medications in RA by language or education level.

Of note, LEP patients had better knowledge on the alcohol-consumption question. This may be a result of clinicians spending more time explaining certain risk factors to patients with language barriers or simply lower alcohol consumption among those non-English-proficiency subjects in this study (although the question related to knowledge, not behavior). LEP was not statistically significantly correlated with poor knowledge on birth defects or with the two numeracy questions. The association of LEP with poorer overall knowledge emphasizes the need to first identify language barriers and use professional interpreter services [14] in all RA patients with LEP to ensure the safe and effective use of available medications in this population of patients at high risk of poor outcomes.

Several studies have examined the effect of educational interventions to improve knowledge around methotrexate, with mixed results [7,15]. Burma and colleagues [15] at the University of Iowa showed significant improvement in baseline knowledge of toxicity and safety of methotrexate after a multipronged approach to education, including teaching by a rheumatology nurse and a methotrexate information sheet from the Arthritis Foundation followed by a “MTX pocket card” given to all patients. Our finding of older age being associated with poor knowledge confirms results from this study, which also showed poorer scores for those age 55 and older [7]. A recent smaller study from the United Kingdom (*n* = 51) assessed provider adherence to recommendations for both verbal and written education around methotrexate and patient knowledge, as measured by an MTX knowledge questionnaire. Of the 51 subjects, 94% and 92% had documentation of nurse-led counseling and provision of written materials on MTX, respectively; however, great variation in patient knowledge was noted around the mechanism of action, drug interactions, and side effects. These patients had a slightly higher mean knowledge score (6.3, SD 1.2; of a possible 10) than in our study, but revealed similar results. Significant predictors of better patient knowledge included age and English-language proficiency [15].

Our study has several limitations. The cross-sectional design does not allow inferences regarding causation, although it is unlikely that knowledge about MTX could have affected many of the variables under study. The sample size is relatively small; however, it is unique in the diversity of the subjects. Given that the study was conducted on a population located in an urban area, treated by university-affiliated rheumatologists, we cannot generalize our findings to patients seen in other clinical settings. We were unable to assess formally the extent of education of patients around methotrexate by different clinicians, as this is not standardized across clinics. As of this writing and during the study period, no formal or systematic patient education exists about methotrexate in the two clinics. Another limitation of our study is that we did not evaluate adherence, as some association may occur between knowledge and adherence. The questionnaire used in this study has not been formally validated, although it did show good internal reliability and was based in large part on a prior questionnaire, which reported excellent feasibility of administration and good face and construct validity [7]. The British Society for Rheumatology guidelines regarding intake of alcohol while taking low-dose methotrexate now state that patients may drink alcohol, but limit intake. Therefore, what was considered a “correct” response to the alcohol-intake question in this study may now be considered incorrect by practicing rheumatologists [16].

An important question this study raises is, how can we best make safer this highly effective and inexpensive therapy for RA? Methotrexate is a known teratogen, and although this fact was known by the great majority of women of childbearing age in our sample, fewer than a fourth of the men were aware of this fact. The true incidence of pregnancy among women taking methotrexate is not known, but a case series of pregnancies in women with autoimmune diseases in which the fetus was exposed to methotrexate *in utero* revealed congenital abnormalities in 17%, as contrasted with a rate of 2% to 3% in the general public [17]. Discontinuation of methotrexate due to side effects has been shown to be twice as high among patients with active RA who were not taking folic acid as compared with those who were taking 1 mg daily [5]. A meta-analysis of studies from the prebiologic era did report a significant association of heavy alcohol intake and liver fibrosis on biopsy among subjects taking methotrexate [6]. If we educate patients with RA on risks to the fetus, importance of folic acid, and alcohol-related liver toxicity, they may be more likely to comply with the medication regimen, its use will be safer, and society will save money, as patients may avoid having to move onto more expensive biologic use and have improved disease activity, greater function, and less work loss. If formal-education interventions are put in place, it will be imperative to measure the effects of such interventions on adherence, safety, and health outcomes, such as function.

Although we hypothesized an association between limited English proficiency and poor methotrexate knowledge, the findings that better function and biologic use were associated with poor knowledge are more unexpected. One could hypothesize that subjects with poorer function are more attuned to the importance of their medication, have more difficulty treating their RA, and have been exposed to more combinations of therapies, and thus, exposed to more teaching and explanation of risks and benefits by their clinicians. With regard to biologic use, it is possible that either these subjects or the prescribing clinicians focus more on the risks and benefits of the biologic and place less emphasis on methotrexate in the clinical setting. It may also be a reflection of polypharmacy and the challenge of conveying or retaining key pieces of information on each medication.

## Conclusions

Among a diverse sample of adults with RA, the median score on a methotrexate questionnaire was 5 of 10, indicating low overall knowledge. Older age, lack of education, limited English-language proficiency, biologic use, and higher function (as measured by the HAQ) were statistically significantly correlated with poor knowledge. Identification of language barriers and improved clinician-patient communication around methotrexate dosing, side effects, and teratogenicity are necessary to ensure safety and maximal benefit of one of the most commonly used medications to treat RA. The use of professional interpreter services for all RA patients who lack English-language proficiency and interventions that include plain language educational materials in multiple languages must be developed to guarantee effective, safe, and high-quality patient-centered care.

## Abbreviations

ACR: American College of Rheumatology; AOR: Adjusted odds ratio; BA: Bachelor of arts; DMARD: Disease-modifying antirheumatic drug; EP: English proficient; ETOH: alcohol; HAQ: Health assessment questionnaire; HS: High school; LEP: Limited English proficient; MTX: Methotrexate; RA: Rheumatoid arthritis; UCSF: University of California, San Francisco.

## Competing interests

The authors report no competing interests.

## Authors’ contributions

JB, LT, JI, JG, DS, and EY contributed to the study design. JB, LT, JI, JG, and EY contributed to acquisition of data. JB, GS, LT, DS, and EY contributed to analysis and interpretation of data. JB, GS, LT, JI, JG, DS, and EY contributed to manuscript preparation and revision. JB, LT, GS, JI, JG, DS, and EY have given final approval of the submitted manuscript.

## Supplementary Material

Additional file 1 **Methotrexate knowledge questionnaire.** This questionnaire includes 11 multiple-choice and true/false questions about methotrexate as well as two items (multiple choice) that test numeracy related to methotrexate dosing. The 135 subjects completed the questionnaire, and the number (%) of those who responded to each option is listed to the right of each response. Click here for file
