# Supplementary material for: Poor knowledge of methotrexate associated with older age and limited English-language proficiency in a diverse rheumatoid arthritis cohort
Source: Arthritis Res Ther. 2013 Oct 22;15(5):R157. doi: 10.1186/ar4340 (PMC3978686; doi:10.1186/ar4340)
Supplement: Additional file 1 — Methotrexate knowledge questionnaire. This questionnaire includes 11 multiple-choice and true/false questions about methotrexate as well as two items (multiple choice) that test numeracy related to methotrexate dosing. The 135 subjects completed the questionnaire, and the number (%) of those who responded to each option is listed to the right of each response. [file ar4340-S1.doc]

| **Appendix : Methotrexate knowledge questionnaire and percentage correct on each question, UCSF RA Cohort (n=135)** | N (%) |
| --- | --- |
| 1. How often is methotrexate usually taken? |  |
| a. once a day | 8 (6) |
| b. three times a week | 5 (4) |
| c. one day a week | 108 (80)* |
| d. every other day | 0 |
| e. don't know | 14 (10) |
|  |  |
| 2. When you are taking methotrexate, how much alcohol are you allowed to drink? |  |
| a. five drinks per week | 2 (2) |
| b. one drink per week | 8 (6) |
| c. no limit | 0 |
| d. none | 72 (53)* |
| e. don't know | 53 (39) |
|  |  |
| 3. True/False: As long as you are taking methotrexate, you should have blood tests at least every 2-3 months. |  |
| a. True | 114 (84)* |
| b. False | 6 (5) |
| c. don't know | 15 (11) |
|  |  |
| 4a. (for women <50, n=37) T/F: Methotrexate can cause birth defects if a woman is taking it at the time she becomes pregnant, or if she takes it during pregnancy. |  |
| a. True | 30 (81)* |
| b. False | 7 (19) |
|  |  |
| 4b. (for men, n=23) T/F: Methotrexate can cause birth defects if a man is taking at the time he causes a woman to become pregnant. |  |
| a. true | 5 (22)* |
| b. false | 18 (78) |
|  |  |
| What are the possible side effects of methotrexate? Answer yes to all that apply. |  |
| 5. Nausea | 57 (42)* |
| 6. Low blood counts | 36 (27)* |
| 7. Mouth sores | 58 (43)* |
| 8. Allergic reaction in lungs | 31 (23)* |
| 9. Liver problems | 77 (57)* |
|  |  |
| 10. T/F: Folic acid helps to reduce side effects of methotrexate. |  |
| a. True | 87 (64)* |
| b. False | 48 (36) |
|  |  |
| 11. Which one of the following statements is true about how methotrexate works in rheumatoid arthritis? |  |
| a. It is a quick-acting pain pill | 7 (5) |
| b. It helps to slow down damage to joints | 87 (64)* |
| c. It fights infection | 5 (4) |
| d. It thins the blood | 0 |
| e. don't know | 36 (27) |
|  |  |
| 12. Let's say you take eight 2.5mg tablets every Sunday. How many milligrams would you take per week? |  |
| a. 2.5mg | 4 (3) |
| b. 8mg | 0 |
| c. 10mg | 3 (2) |
| d. 20 mg | 89 (66)* |
| e. don't know | 39 (29) |
|  |  |
| 13. Let’s say you take six 2.5mg tablets every Sunday but take half the amount in the morning and half in the evening. How many milligrams would you take in the morning? |  |
| a. 2.5mg | 4 (3) |
| b. 6mg | 6 (4) |
| c. 7.5 mg | 81 (60)* |
| d. 15 mg | 8 (6) |
| e. don't know | 36 (27) |

*Asterix indicates the correct response.
